# The association between apolipoprotein E and gallstone disease: an updated meta-analysis

**DOI:** 10.1186/s12881-019-0843-6

**Published:** 2019-06-14

**Authors:** Lizhuo Li, Xin Qiao, Xia Wang, Di Liu, Qingmu Xue, Lu Han, Fei Dai, Guomin Ma, Zhipeng Yang, Tao Zhang, Shuo Yang, Shikang Cai, Mingyue Gao, Jingyun Yang

**Affiliations:** 1 Emergency Department, Xuanwu Hospital, Capital Medical University, Beijing, China; 2 Department of Critical Care and Emergency Medicine, The First Affiliated Hospital of Hainan Medical University, Haikou, Hainan China; 3 Department of Animal Laboratory, School of Basic Medical Sciences, Capital Medical University, Beijing, China; 4 Department of Physiology and Pathophysiology, School of Basic Medical Sciences, Capital Medical University, Beijing, China; 5 Beijing Key Laboratory of Clinical Epidemiology, School of Public Health, Capital Medical University, Beijing, China; 6 School of Basic Medicine, Shenyang Medical College, Shenyang, Liaoning China; 7 Division of Gastroenterology, The Second Affiliated Hospital of Xi’an Jiaotong University, Xi’an, Shaanxi China; 8 Department of Radiology, Liaoning Provincial People’s Hospital, Shenyang, Liaoning China; 9 The First Research Institute, Ministry of the Public Security, Beijing, China; 10 Department of Epidemiology and Health Statistics, Shenyang Medical College, Shenyang, Liaoning China; 11 Hainan Medical University, Haikou, Hainan China; 12 Division of Statistics, School of Economics, Shanghai University, Shanghai, China; 13 Research Center of Financial Information, Shanghai University, Shanghai, China; 14 Rush Alzheimer’s Disease Center, Rush University Medical Center, Chicago, IL USA; 15 Department of Neurological Sciences, Rush University Medical Center, Chicago, IL USA

**Keywords:** *APOE*, Gallstone disease, Polymorphism, Meta-analysis

## Abstract

**Background:**

Gallstone disease (GSD) is a common biliary tract disease worldwide. Previous studies have investigated the association of *apolipoprotein E (APOE) E4* with GSD and reported inconsistent results.

**Methods:**

In this paper, we conducted meta-analyses to examine whether APOE E4 is associated with the risk of GSD. A systematic literature search was performed in PubMed, Cochrane Library, EMBASE, and Google Scholar using the following inclusion criteria: 1) Studies on human subjects; 2) subjects in the control group must undergo ultrasound GSD screening, and presence of GSD in the experiment group can be clearly determined, e.g., diagnosis of GSD through ultrasound screening or a previous history of cholecystectomy or cholelithiasis; 3) the studies reported *APOE* genotype data (APOE E4+ vs. E4-) for subjects with and without GSD. In all the meta-analyses, we used random-effects models to calculate the odds ratios (ORs) as a measure of association as well as the corresponding confidence intervals (CIs).

**Results:**

Our literature search found 13 publications with 14 studies, including a total of 1632 GSD patients and 5001 controls, that met the eligibility criteria and were included in the meta-analyses. We did not find a significant association between *APOE* E4 and risk of GSD (OR = 1.23, 95% CI: 0.89–1.68; *p* = 0.205). No significant associations were observed in subgroup analyses by gender and mean age. We obtained similar insignificant findings if an additive model was used, if subjects who had E2E4 genotype were excluded, or if low-quality studies were excluded.

**Conclusion:**

Our meta-analysis found insufficient evidence for the effect of *APOE* E4 on GSD risk. Future studies with large sample sizes that control for important confounding/risk factors are needed to validate our findings and to explore other genetic loci that might affect GSD risk.

**Electronic supplementary material:**

The online version of this article (10.1186/s12881-019-0843-6) contains supplementary material, which is available to authorized users.

## Background

Gallstone disease (GSD) is one of the most prevalent biliary tract diseases worldwide [1], affecting 10–15% of the adult population in the United State [2]. Among gastrointestinal problems, GSD is a leading cause for hospital admissions, with an estimated 1.8 million ambulatory care visits each year [3]. GSD constitutes a major burden to the health care systems, with an annual cost of around $6.5 billion in the USA [2].

There are two major types of gallstones: cholesterol stones, which mainly consistent of cholesterol monohydrate crystals and precipitates of amorphous calcium bilirubinate, and pigment stones, which mainly contain calcium bilirubinate. The exact pathogenesis of GSD remains to be determined, and efficient strategies for primary prevention and nonsurgical therapies are still under development.

The etiology of GSD is multifactorial and involves interaction of genetic and environmental factors. Previous research has identified multiple risk factors for the development of GSD, such as age [4, 5], female gender [6], obesity [7], and diabetes mellitus [8]. Twin research indicated that the heritability of GSD was approximately 25% [9]. Meanwhile, multiple genes have been reported to be associated with increased GSD risk, such as ATP Binding Cassette Subfamily G Member 8 (*ABCG8*) [10], mucin-like protocadherin (*MUPCDH*) [11] and apolipoprotein E (*APOE*) [12].

The *APOE* gene is located on chromosome 19. *APOE* is a major component of very low-density lipoproteins (VLDLs), which is critical for removing excessive blood cholesterol and maintaining normal cholesterol level. Defects in *APOE* gene in human can lead to familial type III hyperlipoproteinemia (HLP III) showing impaired clearance of chylomicron, VLDL, LDL and increased blood cholesterol [13]. *APOE* has 3 polymorphic alleles, E2 (cys112, cys158), E3 (cys112, arg158), and E4 (arg112, arg158). The E4 has been found to be implicated in multiples diseases/disorders, such as impaired cognition, late-onset Alzheimer’s Disease, and ischemic cerebrovascular disease [14, 15].

Human and mouse model studies have been conducted to examine the role of *APOE* in the development of GSD. Research with *APOE*-deficient mice showed decreased gallstone formation compared to the wild-type mice, suggesting a role of *APOE* in gallstone formation [16]. However, findings in the human regarding the role of *APOE* in GSD formation are inconsistent. For example, a positive association was found between *APOE* E4 genotype and cholesterol crystals in bile, fast cholesterol crystallization in gallbladder bile and a higher cholesterol content in gallstones [12, 17]. However, other studies failed to confirm the findings [18, 19]. Moreover, presence of E4 allele was found to be an independent factor enhancing gallstone clearance in patients undergoing extracorporeal shock-wave lithotripsy (ESWL), but E4 carriers showed a higher recurrence rate following ESWL [20].

Previous studies also examined the relationship between *APOE* polymorphisms and GSD risk, with inconsistent conclusions. To the best of our knowledge, two meta-analyses have been conducted to address the relationship between *APOE* and GSD risk [21, 22]. The former one was included in a paper published in 2013, focusing on the association of eight genetic variants with GSD using a Mendelian randomization approach. This meta-analysis included studies published up to 2012. Some publications were missed in the literature search. The latter one was published in 2012, and included 17 studies with a total of 1773 cases and 2751 controls. This meta-analysis suffers from several methodological concerns, as outlined in more detail in the discussion section. Moreover, new studies appeared after the two meta-analyses. Therefore, to better examine the association of *APOE* genotype with GSD risk, we performed this updated meta-analysis which adapted more stringent criteria regarding inclusion of eligible studies and included most recent publications.

## Methods

As our study used a systematic review and meta-analysis, ethical approval of this study and informed consent statement are not required.

### Eligibility criteria

The following inclusion criteria were used to determine study eligibility: 1) Studies on human subjects; 2) subjects in the control group must undergo ultrasound GSD screening, and presence of GSD in the experiment group can be clearly determined, e.g., diagnosis of GSD through ultrasound screening or a previous history of cholecystectomy or cholelithiasis; 3) the studies reported *APOE* genotype data (*APOE* E4^+^ vs. E4^−^) for subjects with and without GSD. We chose the one with a larger sample size if multiple studies used overlapping data.

### Search strategy

Two authors (LL and JY) performed an extensive literature search in PubMed, Cochrane Library, EMBASE and Google Scholar for papers published before July 18, 2017. The keywords used in the literature search are provided online (Additional file 1: Keywords used in the literature search).

We retrieved all potentially relevant publications to evaluate study eligibility. We also searched the references in all relevant studies for research that might have been missed during the literature search. The two authors performed the literature search independently. The search was limited to studies published in English. Any disagreement was resolved by group discussion (LL, XQ and JY).

### Data extraction

Two authors (LL and JY) independently extracted the following data from the eligible studies, according to a pre-specified protocol for data extraction: name of the first author, year of publication, participants characteristics including sample size, mean age, distribution of gender, race/country of origin of the participants, diagnosis of GSD, and *APOE* genotype data for patients with and without GSD. Any discrepancies were resolved in a group meeting. Quality of the included studies were assessed by two authors (LL and JY) independently using Newcastle–Ottawa scale (NOS) [23]. Extracted data were entered into a computerized spreadsheet for analyses.

### Data analysis

All studies satisfied Hardy–Weinberg equilibrium (HWE) regarding the genotype in the control group. Odds ratios (ORs) were used as a measure of association between the *APOE* genotype and GSD risk. We used random-effects models to calculate the ORs and the corresponding 95% confidence intervals (CIs) in all the meta-analyses. Between-study heterogeneity was assessed using I^2^, and publication bias was evaluated using a funnel plot and Egger’s test.

### Sensitivity/additional analysis

We examined the association by gender, and repeated the analysis by excluding subjects who had E2E4 genotype, and subsequently examined the association using an additive model (e.g., carrying one or two E4 alleles vs. no E4 allele). Finally, we repeated the analysis by excluding studies of low quality (NOS < 6 stars).

All statistical analyses were performed using Stata 11.2 (StataCorp LP, College Station, TX, USA) and SAS version 9.4 (SAS Institute Inc., Cary, NC, USA). A *p* < 0.05 was considered statistically significant. This study was reported according to the PRISMA guidelines [24].

### Availability of data and materials

No additional data are available.

## Results

### Study selection and characteristics

**Fig. 1:**
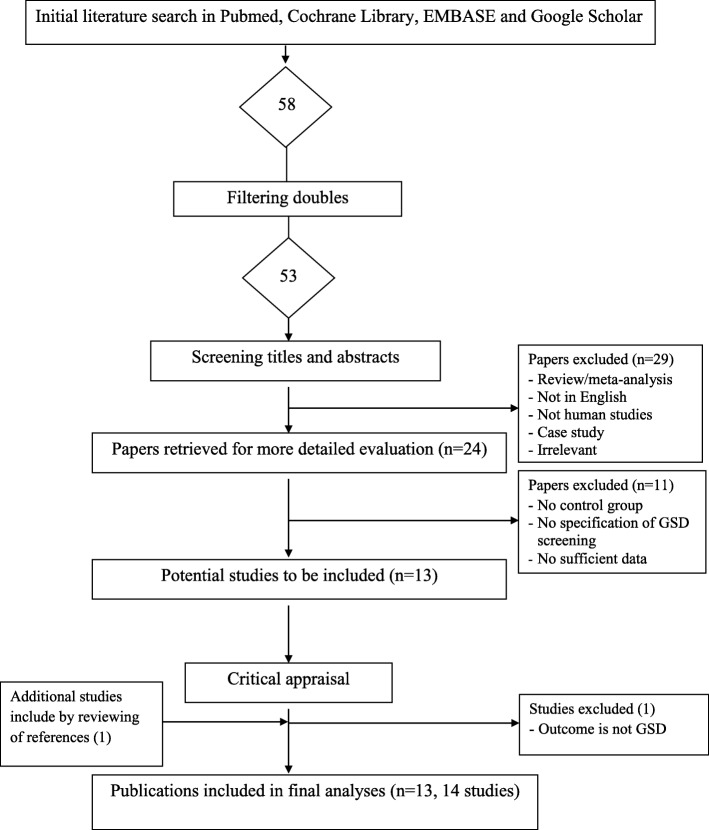
Flow diagram of the selection process of the studies included in the meta-analyses. Note: Please see the Methods section for additional details

Figure 1 shows the selection of eligible studies included in our meta-analyses. We identified a total of 53 potential publications through our initial search. After screening of the abstracts, 29 publications were excluded either because they were not about human subjects, were not in English, were case studies, were reviews or meta-analyses, or were irrelevant. This left 24 studies which were retrieved for more detailed evaluations. We excluded an additional 11 studies because there was no control group, or the studies did not specify GSD screening in the control group or did not provide sufficient data. This led to 13 potentially relevant publications for our analysis. We identified one more study through searching the references of the potential studies, and then excluded one more study because the outcome is not GSD. This led to 13 publications with 14 studies that met the eligibility criteria and were included in our analyses [12, 21, 25–36].

All included publications had been published since 1994. Basic characteristics of the included studies were presented in Table 1. Most of the studies are of good quality. The combined study included 1632 GSD patients and 5001 subjects without GSD.

**Table 1:** Basic characteristics of all the studies included in the meta-analyses

Study	Year of publication	Country/origin	GSD			Control			Diagnosis of GSD	NOS
			n	Age	Male (%)	n	Age	Male (%)		
Rollan et al. [25]	1994	Chile	109	48 ± 13	42.2	110	37 ± 12	43.6	US	5
Bertomeu et al. [12]	1996	Spain	160	59 ± 12.6	34.4	125	58 ± 11.2	34.4	CG/US	8
Niemi et al. [26]	1999	Finland	148	53	31.8	896	51.2	52.8	US	9
Ko et al. [27]	2000	USA	52	24.4 ± 4.4	0	104	25.2 ± 4.6	0	US	8
Abu et al. [28]	2002	Israel	10	–	–	124	–	–	US	7
Hasegawa et al. [29]	2003	Japan	79	55 ± 8.9	46.8	53	39 ± 7.3	60.4	VS/infrared	6
Jiang et al. [30]	2004	China	105	47.5 ± 11.0	74.3	274	47.9 ± 12.2	67.2	US	6
Dixit et al.^a^ [31]	2006	India	207	44.7 ± 13.2	32.2	322	44.0 ± 11.5	36.0	US	7
Mella et al. [32]	2007	Chile	117	49 ± 12	–	122	40 ± 13	–	US	7
Mella et al. [32]	2007	Germany	184	63 ± 13	–	184	63 ± 13	–	US	7
Jaime et al. [33]	2010	Mexico	101	51.9 ± 11.2	13.9	101	51.7 ± 11.0	13.9	US	7
Pinheiro-Júnior et al.^b^ [34]	2012	Brazil	107	46.6 ± 11.2	17.5	104	40.6 ± 9.7	20	US	7
Martinez-Lopez et al. [35]	2015	Mexico	90	40.6 ± 13.8	8	371	37.1 ± 11.5	–	US	7
Shabanzadeh et al.^c^ [36]	2017	Denmark	162	60	34.1	2112	40	52	US	7

Data for age were mainly presented as mean ± SD, or as median (range)

^a^ Data for age and gender for the GSD group were based on 214 patients with GSD

^b^ Data for age and gender were based on 114 subjects with cholelithiasis and 106 subjects without cholelithiasis

^c^ Data for age and gender were based on 504 subjects with GSD and 4992 subjects without GSD. Data for age represent the median age of the GSD group and the control group, respectively

*CG* cholecystogram, *Chole* cholecystectomy, *GSD* gallstone disease, *NDCD* the National Danish Causes of Death, *NDPR* the National Danish Patient Registry, *SD* standard deviation, *US* ultrasound, *VS* visual inspection in cholecystectomy or liver transplantation

### Assessment of publication Bias

**Fig. 2:**
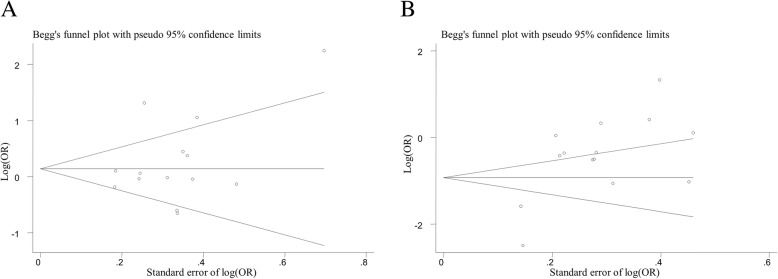
Funnel plot for meta-analysis of the association of *APOE* E4 with GSD risk. **a** Funnel plot for meta-analysis if *APOE* E4 (yes vs. no); **b**) Funnel plot for meta-analysis of *APOE* E4 assuming an additive model. The x-axis is the standard error of the log-transformed odds ratio (log [OR]), and the y-axis is the log-transformed odds ratio. The horizontal line in the figure represents the overall estimated log-transformed odds ratio. The two diagonal lines represent the pseudo 95% confidence limits of the effect estimate. GSD, gallstone disease; OR, odds ratio

We did not find evidence of a significant publication bias for the meta-analysis of the 14 included studies (*p* = 0.338; Fig. 2a), or for the meta-analysis excluding subjects who had E2E4 allele (*p* = 0.483). There was no evidence of publication bias in stratified meta-analysis by gender, or by mean age (all *p* > 0.190). We found evidence of a publication bias for the meta-analysis using an additive model (*p* = 0.005; Fig. 2b).

### Association of APOE with GSD

**Fig. 3:**
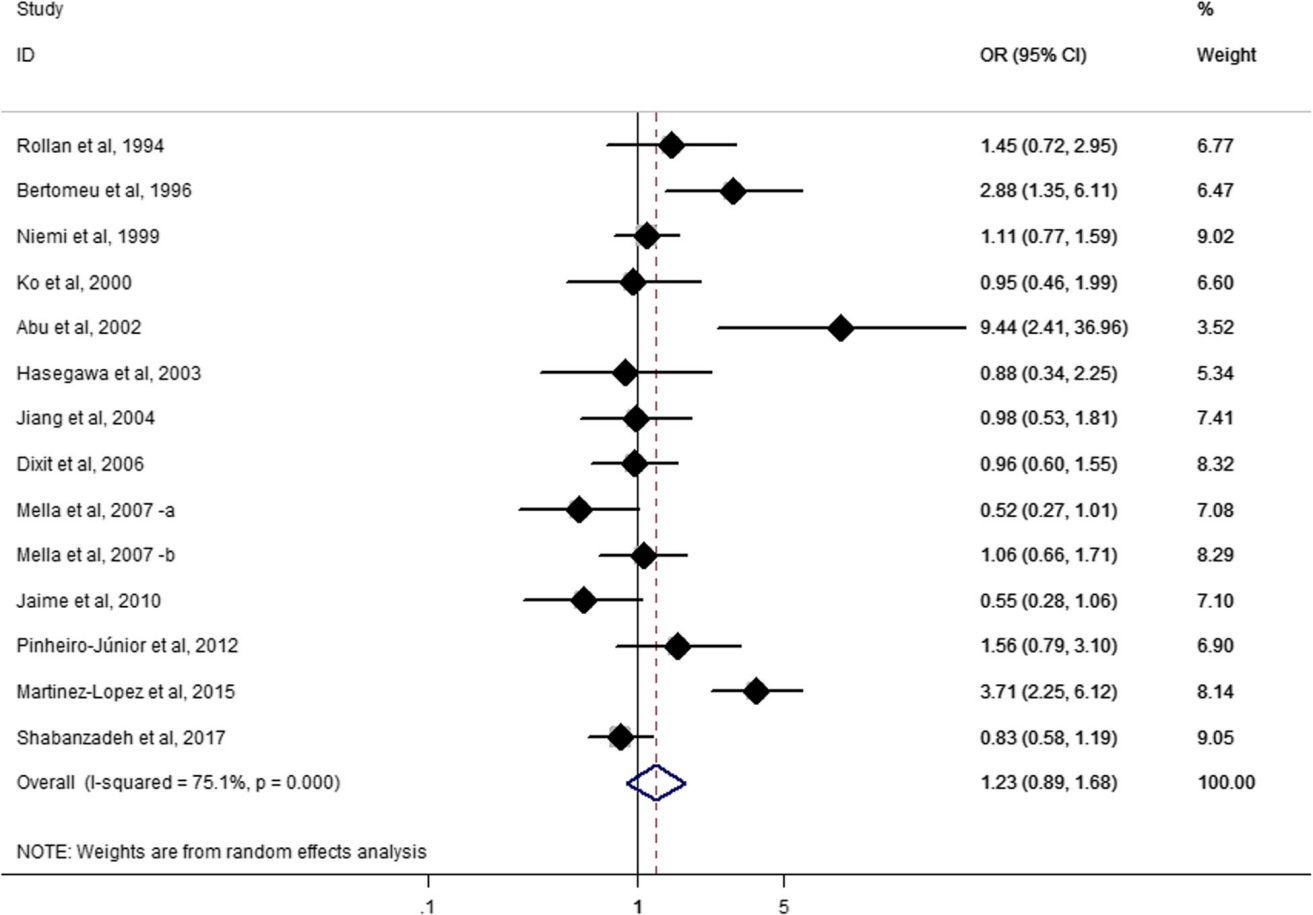
Forest plot for meta-analysis of the association of *APOE* E4 with GSD risk. Each study is represented by a square whose area is proportional to the weight of the study. The overall effect from meta-analysis is represented by a diamond whose width represents the 95% CI for the estimated odds ratio (OR). GSD, gallstone disease; OR, odds ratio; CI, confidence interval

We found no association of *APOE* E4 with the risk of GSD in the meta-analysis including all the 14 studies (OR = 1.23, 95% CI: 0.89–1.68; *p* = 0.205; Fig. 3). There was high heterogeneity among the included studies (I^2^ = 75.1%, *p* < 0.0001). We found no association in either the male (OR = 1.32, 95% CI: 0.77–2.27; *p* = 0.317; I^2^ = 20.3%, p for heterogeneity = 0.285), or the female subjects (OR = 1.17, 95% CI: 0.77–1.77; *p* = 0.474; I^2^ = 40.5%, p for heterogeneity = 0.169).

We then excluded subjects who had E2E4 genotype, and found no significant association of *APOE* E4 with GSD risk (OR = 1.30, 95% CI: 0.90–1.88; *p* = 0.156; I^2^ = 75.2%, p for heterogeneity< 0.0001). We then assessed the association assuming an additive genetic model, and found no statistically significant dosage effect of *APOE* E4 allele on the risk of GSD (OR = 0.63, 95% CI: 0.36–1.12; *p* = 0.114; I^2^ = 94.7%, p for heterogeneity< 0.0001).

We also divided the included studies into two subgroups based on the mean age in the control group: > 50 and ≤ 50 years, and conducted corresponding subgroup analysis. We found no statistically significant association of *APOE* E4 in both the > 50 age group (OR = 1.13, 95% CI: 0.68–1.88; *p* = 0.630; I^2^ = 71.7%, p for heterogeneity = 0.014), and in the ≤50 age group (OR = 1.14, 95% CI: 0.77–1.69; *p* = 0.521; I^2^ = 75.3%, p for heterogeneity < 0.0001). We repeated the analysis by excluding studies of low quality (NOS < 6 stars). Our findings remain essentially unchanged (OR = 1.21, 95% CI: 0.87–1.70; *p* = 0.257; I^2^ = 76.8%, p for heterogeneity< 0.0001). Finally, five studies (four publications) provided data regarding association of *APOE* genotype with cholesterol GSD [29, 32–34]. We performed meta-analysis using data from these five studies, and got similar non-significant results (OR = 0.84, 95% CI: 0.56–1.27; *p* = 0.418; I^2^ = 47.8%, p for heterogeneity = 0.105).

## Discussion

In this paper, we performed a systematic literature search and conducted meta-analyses to examine the association of *APOE* with GSD. In the pooled analysis of 1632 GSD patients and 5001 subjects without GSD, we did not find evidence for significant association of *APOE* with GSD risk. Similar non-significance was observed in subgroup analysis by gender and mean age, and in other sensitivity analyses.

**Fig. 4:**
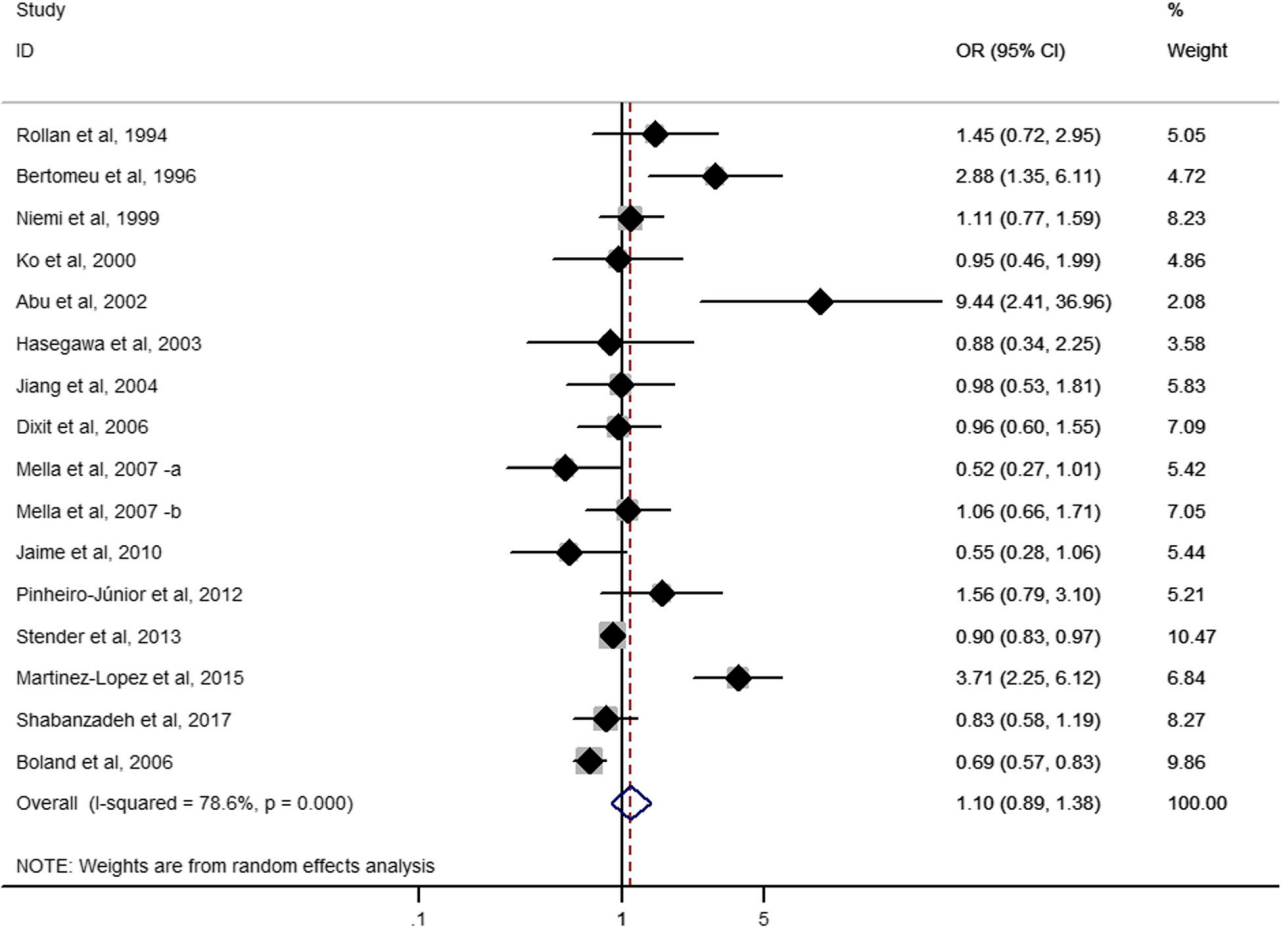
Forest plot for meta-analysis of the association of *APOE* E4 with GSD risk after including the study by Boland et al. Each study is represented by a square whose area is proportional to the weight of the study. The overall effect from meta-analysis is represented by a diamond whose width represents the 95% CI for the estimated odds ratio (OR). GSD, gallstone disease; OR, odds ratio; CI, confidence interval

A previous meta-analysis published in 2012 of 17 studies from 16 publications examined the association of *APOE* E2/E3/E4 polymorphisms with GSD risk [22]. However, the main findings of this study are misleading due to the several methodological issues. The authors claimed that comparison of alleles E4 with E3 yielded a 25% increased risk that was statistically significant (*p* = 0.0003). However, based on the forest plot and the 95% CI (0.97–1.61), this increased risk should not be statistically significant (actual *p* = 0.084 based on the CI). Similar mistakes (wrong calculation of the *p*-values) can be found in the other findings throughout the publication. This meta-analysis also included one study which provided genotype frequency for cholesterol gallstone patients and pigment stone patients [37]. Out of the 16 publications included in the study, seven were based on Chinese subjects. After excluding these seven studies, no significant association was found between *APOE* E4 and GSD risk in non-Chinese studies. Our updated meta-analysis included publications missed by the previous meta-analysis, and we only retained studies in which presence/absence of GSD can be relatively accurately determined. We did not include two studies despite their relative samples sizes because in one study, the existence of GSD at baseline was determined according to a phone interview of medical history [38], and in the other study, subjects in the control group did not undergo GSD screening [21]. Including such studies could bias the results as subjects may have asymptomatic GSD. Nonetheless, we performed a sensitivity analysis by including the two studies in the meta-analysis, and obtained similar findings (OR = 1.10, 95% CI: 0.89–1.38; *p* = 0.376; I^2^ = 78.6%, p for heterogeneity< 0.0001; Fig. 4).

Out of the 14 studies included for meta-analyses in this paper, only three studies reported a significantly increased risk of GSD in *APOE* E4 carriers [12, 28, 35]. while other studies reported no significant association. It is also interesting to note that some studies seemed to indicate a trend of protective effect of *APOE* E4 on GSD risk. For example, the study by Jaime et al. [33] found that the risk of GSD decreased by 45% among *APOE* E4 carriers, compared to non-carriers (OR = 0.55, 95% CI: 0.28–1.06; *p* = 0.073). Similar findings held when we excluded E2E4 carriers. Selection bias may be underlying the inconsistent findings. In the three studies reporting a positive association, the *APOE* E4 allele frequency is relatively low in the control group (4–8%) compared to the general population [39].

Our study had several limitations: Although efforts were made in the systematic literature search in an attempt to include as many eligible studies as possible, the pooled sample size is still small. We had to exclude two large studies because the presence/absence of GSD cannot be clearly determined. More studies with larger samples are needed to further validate our findings. The heterogeneity was high for many of the meta-analyses in this study. Additional data of participants for each individual study were limited, and were only available for some of the included studies, making it hard to identify the real sources of heterogeneity. To explore the possible sources of heterogeneity, we performed a random-effects meta-regression analysis by including age, gender and race. However, none of the three variables were statistically significant, and there were 59.4% remaining residual variation due to heterogeneity. Interestingly, we obtained acceptable heterogeneity in a sensitivity analysis including 5 studies on the association of *APOE* with risk of cholesterol GSD. We got a similar non-significant finding, further supporting that there was no association between cholesterol gallstones and *APOE* E4 genotype. As in other meta-analyses that only utilized published data, we could not control for potential confounding/risk factors, such as age, sex [40], ethnicity and dietary pattern [41]. The estimated effect of *APOE* on GSD risk might be greatly confounded by such factors, and therefore could influence the validity of any meta-analysis that uses unadjusted results. Therefore, such important confounding factors should be taken into account in future studies on the relationship between *APOE* and the GSD risk.

## Conclusions

We performed meta-analyses to examine the association of *APOE* E4 with GSD. We found no significant effect of *APOE* E4 on GSD risk. Future studies with large sample sizes that control for important confounding risk factors are needed to validate our findings and to explore additional genetic loci that might affect GSD risk. Prospective studies that take into account important comorbid factors, such as hypertension, diabetes, and coronary artery disease, are also needed to fully elucidate the relationship between *APOE* E4 and GSD risk.

## Additional file

Additional file 1: Keywords used in the literature search. Combination of keywords used in the literature search of potential publications in PubMed, Cochrane Library, EMBASE and Google Scholar (DOCX 14 kb)

## Data Availability

Raw data can be accessed from the corresponding author on reasonable request.

## Abbreviations

ABCG8: ATP Binding Cassette Subfamily G Member 8
APOE: Apolipoprotein E
CI: Confidence interval
GSD: Gallstone disease
HLP III: Type III hyperlipoproteinemia
MUPCDH: Mucin-like protocadherin
OR: Odds ratio
VLDL: Very low-density lipoproteins
