# Supplementary material for: The association between apolipoprotein E and gallstone disease: an updated meta-analysis
Source: BMC Med Genet. 2019 Jun 14;20:109. doi: 10.1186/s12881-019-0843-6 (PMC6570961; doi:10.1186/s12881-019-0843-6)
Supplement: Supplementary file 1 — Keywords used in the literature search. Combination of keywords used in the literature search of potential publications in PubMed, Cochrane Library, EMBASE and Google Scholar (DOCX 14 kb) [file 12881_2019_843_MOESM1_ESM.docx]

**Key words used in the literature search**

**Pubmed**

(APOE[Title/Abstract] OR Apolipoprotein E[Title/Abstract]) AND (gallstone[Title/Abstract] OR biliary stone[Title/Abstract] OR bile duct stone [Title/Abstract] OR cholelithiasis[Title/Abstract])

**Cochrane Library and Embase**

("APOE") and ("gallstone" or "bile duct stone" or "cholelithiasis")

**Google Scholar**

allintitle: apoe gallstone OR biliary OR stone OR bile OR duct OR stone OR cholelithiasis -cholecystectomy
